# 4-Chloro­anilinium hydrogen oxalate hemihydrate

**DOI:** 10.1107/S160053681001144X

**Published:** 2010-03-31

**Authors:** Hajer Rahmouni, Wajda Smirani, Mohamed Rzaigui, Salem S. Al-Deyab

**Affiliations:** a Laboratoire de Chimie des Matériaux, Faculté des Sciences de Bizerte, 7021 Zarzouna Bizerte, Tunisia; b Petrochemical Research Chair, College of Science, King Saud University, Riyadh, Saudi Arabia

## Abstract

In the title hydrated mol­ecular salt, C_6_H_7_ClN^+^·C_2_HO_4_^−^·0.5H_2_O, the water O atom lies on a crystallographic twofold axis. In the crystal, the anions are linked by O—H⋯O hydrogen bonds, forming chains propagating along the *b* axis. These chains are inter­connected through O—H⋯O hydrogen bonds from the water mol­ecules and N—H⋯O hydrogen bonds from the cations, building layers parallel to the *ab* plane.

## Related literature

For background to supra­molecular networks, see: Subramanian & Zawarotko (1994 ▶). For related structures, see: Akriche & Rzaigui (2009 ▶); Dhaouadi *et al.* (2008 ▶).
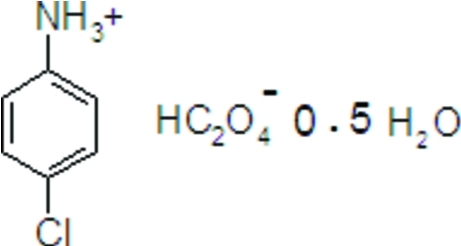


## Experimental

### 

#### Crystal data

C_6_H_7_ClN^+^·C_2_HO_4_^−^·0.5H_2_O
*M* *_r_* = 226.61
Monoclinic, 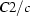

*a* = 26.739 (2) Å
*b* = 5.701 (3) Å
*c* = 13.859 (2) Å
β = 111.02 (3)°
*V* = 1972.0 (11) Å^3^
*Z* = 8
Ag *K*α radiation
λ = 0.56085 Å
μ = 0.20 mm^−1^
*T* = 293 K
0.30 × 0.20 × 0.20 mm

#### Data collection

Enraf–Nonius TurboCAD-4 diffractometer
5686 measured reflections
4806 independent reflections
2341 reflections with *I* > 2σ(*I*)
*R*_int_ = 0.021
2 standard reflections every 120 min  intensity decay: 5%

#### Refinement

*R*[*F* ^2^ > 2σ(*F* ^2^)] = 0.061
*wR*(*F* ^2^) = 0.174
*S* = 1.01
4806 reflections
136 parameters
H-atom parameters not refined
Δρ_max_ = 0.41 e Å^−3^
Δρ_min_ = −0.52 e Å^−3^

### 

Data collection: *CAD-4 EXPRESS* (Enraf–Nonius, 1994 ▶); cell refinement: *CAD-4 EXPRESS*; data reduction: *XCAD4* (Harms & Wocadlo, 1995 ▶); program(s) used to solve structure: *SHELXS97* (Sheldrick, 2008 ▶); program(s) used to refine structure: *SHELXL97* (Sheldrick, 2008 ▶); molecular graphics: *ORTEP-3* (Farrugia, 1997 ▶); software used to prepare material for publication: *WinGX* (Farrugia, 1999 ▶).

## Supplementary Material

Crystal structure: contains datablocks I, global. DOI: 10.1107/S160053681001144X/hb5378sup1.cif

Structure factors: contains datablocks I. DOI: 10.1107/S160053681001144X/hb5378Isup2.hkl

Additional supplementary materials:  crystallographic information; 3D view; checkCIF report

## Figures and Tables

**Table 1:** Hydrogen-bond geometry (Å, °)

*D*—H⋯*A*	*D*—H	H⋯*A*	*D*⋯*A*	*D*—H⋯*A*
O5—H1⋯O1^i^	0.81 (2)	1.97 (2)	2.762 (2)	169 (2)
O3—H3⋯O2^ii^	0.82	1.79	2.606 (2)	173
N1—H1*A*⋯O5	0.89	1.93	2.802 (3)	165
N1—H1*B*⋯O1^iii^	0.89	1.98	2.792 (3)	151
N1—H1*C*⋯O2	0.89	1.92	2.790 (2)	167

Symmetry codes: (i) 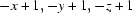; (ii) 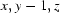; (iii) 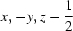.

**Fig. 1.**
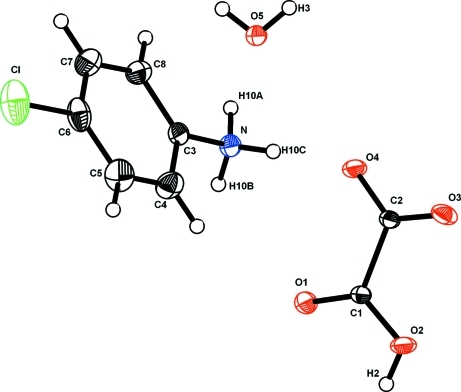
The molecular structure of (I) with displacement ellipsoids for non-H atoms drawn at the 30% probability level.

**Fig. 2.**
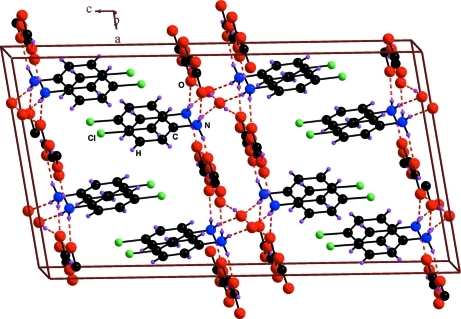
A perspective view of packing of (I).

## supplementary crystallographic information

### Comment

Hydrogen bonding is by far the most well- studied interaction which is employed to control the conformational and topological features of the molecular assembly in the solid state (Subramanian, S. & Zawarotko, J., 1994) . In this paper, we report the synthesis and the X-ray study of the title compound, (I), a new oxalate of para-chloroanilinium hemi-hydrate, C_6_H_7_NCl^+^.HC_2_O_4_^-^.0.5H_2_O. The asymmetric unit contains one oxalate anion, one para-chloroanilinium cation and a water molecule (Fig. 1).

The crystal structure of the title compound is characterized by the existence of inorganic layers, built by HC_2_O_4_^- ^anions, ammonium cations and water molecules. Each anion is connected to its adjacent neighbours by O—H···O strong hydrogen bond to form chains along b axis. These chains are interconnected through O—H···O hydrogen bonds of the water molecules and N—H···O of the ammonium cations to build layers parallel to the (ab) planes at z = 0 and z = 1/2 (Fig. 2).

The protonated *p*-chloroaniline molecule is localized in the interlayer space, and neutralizes the negative charge of the anionic part. These groups are oriented in the same direction forming so intermolecular van der Waals interactions between them and establishing particularly hydrogen bonds with oxygen atoms of the anionic layers.

The C—C, C—O distances and O—C—O, C—C—O angles in oxalate anion have standard values (Akriche, S. & Rzaigui, M., 2009). The examination of the organic molecule shows that the N—C, C—C and C—Cl distances and C—C—C, C—C—N and C—C—Cl angles are comparable with those obtained in other salts associated to the same protonated amine (Dhaouadi, H. *et al.* 2008).

### Experimental

An ethanolic solution of *p*-chloroaniline (50 mmol, in 50 ml) was added under stirring to 100 ml of an aqueous solution of oxalate acid (1 M). Pink blocks of (I) appeared after few days of evaporation at room temperature.

### Refinement

All H atoms were positioned in a difference map and refined on the bond lengths and angles to regularize their geometry [N—H 0.89–0.90, C—H in the range 0.88–0.96 Å (CH_3 _) C—H in the range 0.92–1.00 Å (Ar–H) and O—H in the range 0.87–0.90 Å ] and *U*_iso_(H) [in the range 1.2–1.5 times Ueq of the parent atom]

### Crystal data

**Table:**

C_6_H_7_ClN^+^·C_2_HO_4_^−^·0.5H_2_O	*F*(000) = 936
*M**_r_* = 226.61	*D*_x_ = 1.527 Mg m^−^^3^
Monoclinic, *C*2/*c*	Ag *K*α radiation, λ = 0.56085 Å
Hall symbol: -C 2yc	Cell parameters from 25 reflections
*a* = 26.739 (2) Å	θ = 9–11°
*b* = 5.701 (3) Å	µ = 0.20 mm^−^^1^
*c* = 13.859 (2) Å	*T* = 293 K
β = 111.02 (3)°	Block, pink
*V* = 1972.0 (11) Å^3^	0.30 × 0.20 × 0.20 mm
*Z* = 8	

### Data collection

**Table:**

Enraf–Nonius TurboCAD-4 diffractometer	*R*_int_ = 0.021
Radiation source: fine-focus sealed tube	θ_max_ = 28.0°, θ_min_ = 2.4°
graphite	*h* = −5→44
non–profiled ω scans	*k* = 0→9
5686 measured reflections	*l* = −23→22
4806 independent reflections	2 standard reflections every 120 min
2341 reflections with *I* > 2σ(*I*)	intensity decay: 5%

### Refinement

**Table:**

Refinement on *F*^2^	Primary atom site location: structure-invariant direct methods
Least-squares matrix: full	Secondary atom site location: difference Fourier map
*R*[*F*^2^ > 2σ(*F*^2^)] = 0.061	Hydrogen site location: inferred from neighbouring sites
*wR*(*F*^2^) = 0.174	H-atom parameters not refined
*S* = 1.01	*w* = 1/[σ^2^(*F*_o_^2^) + (0.0711*P*)^2^ + 0.8804*P*] where *P* = (*F*_o_^2^ + 2*F*_c_^2^)/3
4806 reflections	(Δ/σ)_max_ < 0.001
136 parameters	Δρ_max_ = 0.41 e Å^−^^3^
0 restraints	Δρ_min_ = −0.52 e Å^−^^3^

### Special details

**Table:**

Geometry. All esds (except the esd in the dihedral angle between two l.s. planes) are estimated using the full covariance matrix. The cell esds are taken into account individually in the estimation of esds in distances, angles and torsion angles; correlations between esds in cell parameters are only used when they are defined by crystal symmetry. An approximate (isotropic) treatment of cell esds is used for estimating esds involving l.s. planes.
Refinement. Refinement of *F*^2^ against ALL reflections. The weighted *R*-factor wR and goodness of fit *S* are based on *F*^2^, conventional *R*-factors *R* are based on *F*, with *F* set to zero for negative *F*^2^. The threshold expression of *F*^2^ > σ(*F*^2^) is used only for calculating *R*-factors(gt) etc. and is not relevant to the choice of reflections for refinement. *R*-factors based on *F*^2^ are statistically about twice as large as those based on *F*, and *R*- factors based on ALL data will be even larger.

### Fractional atomic coordinates and isotropic or equivalent isotropic displacement parameters (Å^2^)

**Table:**

	*x*	*y*	*z*	*U*_iso_*/*U*_eq_	
H1	0.5152 (9)	0.705 (4)	0.2959 (17)	0.047 (7)*	
Cl1	0.19472 (2)	0.44325 (17)	0.06451 (5)	0.0743 (3)	
O5	0.5000	0.6144 (3)	0.2500	0.0321 (4)	
O2	0.44106 (6)	0.1863 (2)	0.46326 (9)	0.0360 (3)	
C7	0.44211 (7)	0.0194 (3)	0.52200 (12)	0.0249 (3)	
O3	0.44333 (6)	−0.3952 (2)	0.54031 (10)	0.0405 (3)	
H3	0.4408	−0.5227	0.5116	0.061*	
C8	0.43716 (6)	−0.2276 (3)	0.47280 (12)	0.0254 (3)	
O1	0.44687 (6)	0.0354 (2)	0.61417 (9)	0.0401 (3)	
N1	0.42789 (6)	0.2638 (3)	0.25679 (11)	0.0304 (3)	
H1A	0.4460	0.3837	0.2442	0.046*	
H1B	0.4353	0.1338	0.2289	0.046*	
H1C	0.4374	0.2445	0.3248	0.046*	
O4	0.42904 (6)	−0.2531 (2)	0.38251 (9)	0.0389 (3)	
C1	0.37061 (7)	0.3117 (3)	0.21167 (12)	0.0299 (3)	
C6	0.35291 (8)	0.5162 (4)	0.15786 (16)	0.0430 (5)	
H6	0.3773	0.6259	0.1516	0.052*	
C4	0.26302 (8)	0.3917 (5)	0.12275 (15)	0.0463 (5)	
C2	0.33516 (9)	0.1479 (4)	0.22190 (16)	0.0449 (5)	
H2	0.3477	0.0105	0.2588	0.054*	
C5	0.29834 (9)	0.5574 (4)	0.11304 (19)	0.0522 (6)	
H5	0.2857	0.6955	0.0768	0.063*	
C3	0.28077 (9)	0.1887 (5)	0.17710 (18)	0.0523 (6)	
H3A	0.2564	0.0793	0.1838	0.063*	

### Atomic displacement parameters (Å^2^)

**Table:**

	*U*^11^	*U*^22^	*U*^33^	*U*^12^	*U*^13^	*U*^23^
Cl1	0.0367 (3)	0.1244 (7)	0.0581 (4)	0.0117 (3)	0.0125 (2)	−0.0091 (4)
O5	0.0451 (10)	0.0241 (8)	0.0239 (8)	0.000	0.0084 (7)	0.000
O2	0.0605 (8)	0.0192 (5)	0.0290 (6)	−0.0017 (6)	0.0167 (6)	0.0022 (4)
C7	0.0327 (7)	0.0186 (6)	0.0235 (6)	−0.0026 (6)	0.0102 (6)	−0.0021 (5)
O3	0.0757 (10)	0.0167 (5)	0.0308 (6)	−0.0014 (6)	0.0212 (7)	0.0012 (4)
C8	0.0325 (8)	0.0186 (6)	0.0243 (7)	−0.0015 (6)	0.0094 (6)	−0.0005 (5)
O1	0.0714 (9)	0.0255 (6)	0.0286 (6)	−0.0124 (6)	0.0243 (6)	−0.0070 (5)
N1	0.0357 (7)	0.0295 (7)	0.0264 (6)	0.0009 (6)	0.0117 (6)	0.0000 (5)
O4	0.0616 (9)	0.0292 (6)	0.0242 (5)	0.0026 (6)	0.0134 (6)	−0.0036 (5)
C1	0.0354 (8)	0.0305 (8)	0.0235 (7)	0.0009 (7)	0.0102 (6)	−0.0026 (6)
C6	0.0426 (10)	0.0331 (9)	0.0466 (11)	−0.0019 (8)	0.0079 (8)	0.0051 (8)
C4	0.0343 (9)	0.0695 (15)	0.0343 (9)	0.0042 (10)	0.0112 (7)	−0.0095 (10)
C2	0.0476 (11)	0.0448 (11)	0.0454 (11)	−0.0042 (9)	0.0207 (9)	0.0099 (9)
C5	0.0462 (12)	0.0451 (12)	0.0532 (12)	0.0098 (10)	0.0031 (10)	0.0055 (10)
C3	0.0443 (11)	0.0651 (15)	0.0511 (12)	−0.0100 (11)	0.0216 (10)	0.0033 (11)

### Geometric parameters (Å, °)

**Table:**

Cl1—C4	1.736 (2)	N1—H1C	0.8900
O5—H1	0.81 (2)	C1—C6	1.374 (3)
O2—C7	1.2460 (19)	C1—C2	1.373 (3)
C7—O1	1.2407 (19)	C6—C5	1.385 (3)
C7—C8	1.549 (2)	C6—H6	0.9300
O3—C8	1.3055 (19)	C4—C3	1.370 (3)
O3—H3	0.8200	C4—C5	1.376 (4)
C8—O4	1.1996 (19)	C2—C3	1.381 (3)
N1—C1	1.457 (2)	C2—H2	0.9300
N1—H1A	0.8900	C5—H5	0.9300
N1—H1B	0.8900	C3—H3A	0.9300
			
O1—C7—O2	125.89 (15)	C1—C6—C5	119.3 (2)
O1—C7—C8	118.75 (14)	C1—C6—H6	120.3
O2—C7—C8	115.36 (13)	C5—C6—H6	120.3
C8—O3—H3	109.5	C3—C4—C5	121.3 (2)
O4—C8—O3	126.01 (15)	C3—C4—Cl1	119.84 (19)
O4—C8—C7	121.60 (14)	C5—C4—Cl1	118.87 (19)
O3—C8—C7	112.39 (13)	C1—C2—C3	119.6 (2)
C1—N1—H1A	109.5	C1—C2—H2	120.2
C1—N1—H1B	109.5	C3—C2—H2	120.2
H1A—N1—H1B	109.5	C4—C5—C6	119.3 (2)
C1—N1—H1C	109.5	C4—C5—H5	120.3
H1A—N1—H1C	109.5	C6—C5—H5	120.3
H1B—N1—H1C	109.5	C4—C3—C2	119.4 (2)
C6—C1—C2	121.13 (18)	C4—C3—H3A	120.3
C6—C1—N1	119.76 (16)	C2—C3—H3A	120.3
C2—C1—N1	119.09 (16)		

### Hydrogen-bond geometry (Å, °)

**Table:**

*D*—H···*A*	*D*—H	H···*A*	*D*···*A*	*D*—H···*A*
O5—H1···O1^i^	0.81 (2)	1.97 (2)	2.762 (2)	169 (2)
O3—H3···O2^ii^	0.82	1.79	2.606 (2)	173
N1—H1A···O5	0.89	1.93	2.802 (3)	165
N1—H1B···O1^iii^	0.89	1.98	2.792 (3)	151
N1—H1C···O2	0.89	1.92	2.790 (2)	167

Symmetry codes: (i) −*x*+1, −*y*+1, −*z*+1; (ii) *x*, *y*−1, *z*;  (iii) *x*, −*y*, *z*−1/2.
